# Integrating Artificial Intelligence Into Radiation Oncology: Can Humans Spot AI?

**DOI:** 10.7759/cureus.50486

**Published:** 2023-12-13

**Authors:** Nandan M Shanbhag, Abdulrahman Bin Sumaida, Theresa Binz, Syed Mansoor Hasnain, Omran El-Koha, Khalifa Al Kaabi, Mohammad Saleh, Khaled Al Qawasmeh, Khalid Balaraj

**Affiliations:** 1 Oncology/Palliative Care, Tawam Hospital, Al Ain, ARE; 2 Oncology/Radiation Oncolgy, Tawam Hospital, Al Ain, ARE; 3 Oncology/Radiation Oncology, Tawam Hospital, Al Ain, ARE; 4 Radiotherapy Technology, Tawam Hospital, Al Ain, ARE; 5 Radiation Oncology, Tawam Hospital, Al Ain, ARE; 6 Department of Oncology, Tawam Hospital, Al Ain, ARE; 7 Department of Nursing, Tawam Hospital, Al Ain, ARE

**Keywords:** accuracy, efficiency, contouring, limbus ai, radiation oncology, artificial intelligence

## Abstract

Introduction

Artificial intelligence (AI) is transforming healthcare, particularly in radiation oncology. AI-based contouring tools like Limbus are designed to delineate Organs at Risk (OAR) and Target Volumes quickly. This study evaluates the accuracy and efficiency of AI contouring compared to human radiation oncologists and the ability of professionals to differentiate between AI-generated and human-generated contours.

Methods

At a recent AI conference in Abu Dhabi, a blind comparative analysis was performed to assess AI's performance in radiation oncology. Participants included four human radiation oncologists and the Limbus® AI software. They contoured specific regions from CT scans of a breast cancer patient. The audience, consisting of healthcare professionals and AI experts, was challenged to identify the AI-generated contours. The exercise was repeated twice to observe any learning effects. Time taken for contouring and audience identification accuracy were recorded.

Results

Initially, only 28% of the audience correctly identified the AI contours, which slightly increased to 31% in the second attempt. This indicated a difficulty in distinguishing between AI and human expertise. The AI completed contouring in up to 60 seconds, significantly faster than the human average of 8 minutes.

Discussion

The results indicate that AI can perform radiation contouring comparably to human oncologists but much faster. The challenge faced by professionals in identifying AI versus human contours highlights AI's advanced capabilities in medical tasks.

Conclusion

AI shows promise in enhancing radiation oncology workflow by reducing contouring time without quality compromise. Further research is needed to confirm AI contouring's clinical efficacy and its integration into routine practice.

## Introduction

Integrating artificial intelligence (AI) into radiation oncology has been a transformative development, promising to enhance the precision and efficiency of cancer treatments [1]. One of the most critical and time-consuming tasks in radiation therapy planning is delineating target volumes and Organs at Risk (OAR), commonly known as contouring. The advent of AI-based contouring tools, such as the Limbus® AI software, can revolutionize this process by providing rapid and accurate delineations that could significantly impact treatment outcomes [2].

Recent advancements in AI have led to the development of auto-segmentation algorithms capable of delineating anatomical structures with a precision that challenges the expertise of human radiation oncologists. Watkins et al. explored the efficiency gains of unedited AI contours in total marrow irradiation, highlighting the potential of AI to achieve a 100% efficiency gain over traditional methods [3]. This leap in efficiency is not only a testament to the capabilities of AI but also the significance of its role in future oncological practices.

The accuracy of AI contouring is paramount, as the slightest deviation can lead to suboptimal treatment or increased toxicity. A study evaluated the accuracy of cloud-based AI models in contour delineation, suggesting that the effectiveness of AI models is expected to improve with ongoing development, thereby enhancing the reliability of AI-assisted radiation therapy contouring and planning [4].

The impact of AI on clinical efficiency was further demonstrated by another study, which reported that deep learning-based auto-segmentation reduces contouring time and improves clinical efficiency for treating cervical cancer [5]. This finding is particularly relevant as it underscores the potential of AI to streamline workflow in radiation oncology, allowing for more patients to be treated promptly and with greater precision.

A randomized multi-reader evaluation investigated the automated detection and delineation of brain tumors for stereotactic radiosurgery (SRS). This study concluded that deep learning neural networks could be optimally utilized to improve accuracy and efficiency for the clinical workflow in brain tumor SRS, further supporting the integration of AI in complex oncological treatments [6].

The pediatric population also stands to benefit from AI in radiation oncology. A study focused on the auto-segmentation of target volumes and OARs in pediatric cancer patients, emphasizing the importance of accuracy and time efficiency. The study highlighted the potential of AI to safeguard the delicate balance between effective treatment and the preservation of normal tissue, which could affect growth in young patients [7].

The studies above suggest that AI can perform radiation contouring with precision comparable to, if not surpassing, human oncologists. However, the actual test of AI's efficacy lies in its ability to replicate the expertise of human practitioners. The blind prospective comparative analysis in this study conducted at an AI conference co-hosted by the Embassy of the Republic of Korea to the UAE, the Department of Health - Abu Dhabi, and G42 Healthcare, organized by the Korea Health Industry Development Institute (KHIDI), sought to evaluate this very aspect [8].

In this analysis, human radiation oncologists and the AI software Limbus® AI were tasked with contouring the axilla and Internal Mammary Nodal chain from CT scans of a breast cancer patient post-lumpectomy. The results were intriguing; only a small proportion of healthcare professionals and AI experts could identify the AI-generated contours correctly. This challenge in discerning between AI and human-generated contours underscores the sophistication of AI in replicating expert-level medical tasks.

The efficiency of AI in contouring was also tested, with AI completing the task in a fraction of the time taken by human oncologists. This marked efficiency of AI over human counterparts is a promising development, as it suggests that AI can significantly augment the workflow in radiation oncology by reducing time without compromising the contouring quality.

## Materials and methods

A blind comparative analysis was conducted to assess the performance of AI in radiation oncology contouring tasks against that of human experts. The participants for contouring comprised four board-certified radiation oncologists with professional experience ranging from 5 to 15 years and an AI-based contouring software, Limbus® AI. The audience was the delegates at the AI conference co-hosted by the Embassy of the Republic of Korea to the UAE, the Department of Health - Abu Dhabi, and G42 Healthcare, organized by the KHIDI 2023.

De-identified post-lumpectomy breast cancer patient CT scans were sourced. These scans met the necessary resolution and quality standards for radiation therapy planning. Each participant contoured the axillary nodal levels I, II, and III along with the supraclavicular nodal level and the internal mammary nodal levels on the right side. The participant contour was labeled RO 1 to RO 4, denoting the 4 radiation oncologists (ROs), and the AI contour was labeled Limbus® AI. None of the participants could see other contours (single-blinded). The human oncologists performed contouring tasks on an ARIA Somavision workstation (Varian Medical Systems, Inc., Palo Alto, CA, USA) using the contouring workspace version 15.06.56. The AI software, Limbus®, was operated on a Windows 10 PC with a 2 GHz processor and 16 GB RAM. Each participant was tasked with delineating the axilla and internal mammary nodal chain regions from the provided CT scans. The time taken for contour completion was meticulously recorded for each oncologist, starting from the opening of the imaging study to the finalization of the contours. The system automatically logged the Limbus® AI software's processing time.

The contours produced by the human oncologists and the AI were anonymized and randomized for evaluation. An audience of healthcare professionals and AI experts at the “Adopting Digital and AI Technology in Medical Services” conference in Abu Dhabi, co-hosted by the Embassy of the Republic of Korea to the UAE, the Department of Health - Abu Dhabi, and G42 Healthcare, organized by the KHIDI 2023, led a blind review to identify which contours were AI-generated. This assessment was facilitated through an electronic survey utilizing Microsoft® Forms® presenting the contours and corresponding CT images (Figures 6-7 in Appendices). The contours were presented to the conference audience as questions, each with five options. The audience was all given an equal time of five minutes to look at the question and select the appropriate option. This was repeated for the second question. The identification exercise was repeated to assess the potential learning effect with a second set of anonymized contours derived from a different patient's CT scans. The audience was unaware of the initial round's outcomes to prevent bias.

Statistical analysis

Descriptive statistics summarized the demographics of the human oncologists and the audience's composition. The audience's accuracy in identifying AI-generated contours was calculated as a percentage of correct responses. A chi-square test was employed to compare the identification accuracy between the first and second rounds, evaluating the learning effect. The contouring times for human oncologists and the AI were compared using a paired t-test.

Software and hardware specifications

The human oncologists utilized the ARIA Somavision workstation, contouring workspace version 15.06.56, provided by Varian® Medical Systems, Inc., 3100 Hansen Way, Palo Alto, CA 94304-1038, USA. The Limbus® AI Contouring software was run on a Windows® 10 PC with a 2 GHz processor and 16 GB of RAM. Statistical analyses were conducted using data analyses with OpenAI and verified, ensuring all data were processed with rigorous scientific standards.

Ethical considerations

The Institutional Review Board (IRB) at Tawam Hospital reviewed and approved the study protocol with the approval number MF2058-2023-1001. Given the blinded analysis nature of the study and the use of anonymized imaging data, the requirement for informed consent was waived. The human oncologists were unaware of patient-related data and were only presented with the scans.

## Results

Contouring time and volume**

**Figure 1:**
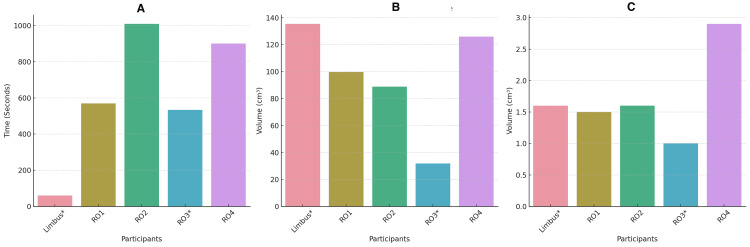
Contouring time and volume A) Contouring time in seconds; B) Supraclavicular and axilla volume in cc; C) Internal mammary nodal volume in cc. RO1: Radiation oncologist 1; RO2: Radiation oncologist 2; RO3*: Radiation oncologist 3; RO4: Radiation oncologist 4; Limbus: Limbus® AI; IMN: internal mammary nodal; cc: cubic centimeters

Limbus® AI demonstrated remarkable efficiency in radiation oncology contouring tasks, significantly outperforming human oncologists in speed. It impressively completed the contouring task in just 60 seconds, highlighting its potential to enhance productivity in this field significantly. Among the human oncologists, RO1 was the quickest, finishing the task in approximately 570 seconds (9 minutes and 30 seconds), closely followed by RO3 in 534 seconds (8 minutes and 54 seconds). It's important to note that RO3 only contoured the supraclavicular area, likely contributing to their relatively faster performance. In comparison, RO4 and RO2 took longer, with RO4 completing the task in around 900 seconds (15 minutes) and RO2 taking the longest at approximately 1009 seconds (16 minutes and 49 seconds) (Figures 1A-1C).

This variability in time taken by the different human oncologists may reflect individual differences in approach, experience, or efficiency, underscoring the diverse methodologies employed by professionals in this field.

Distribution of responses for Question 1: AI contour was Option 1 (correct answer)

**Figure 2:**
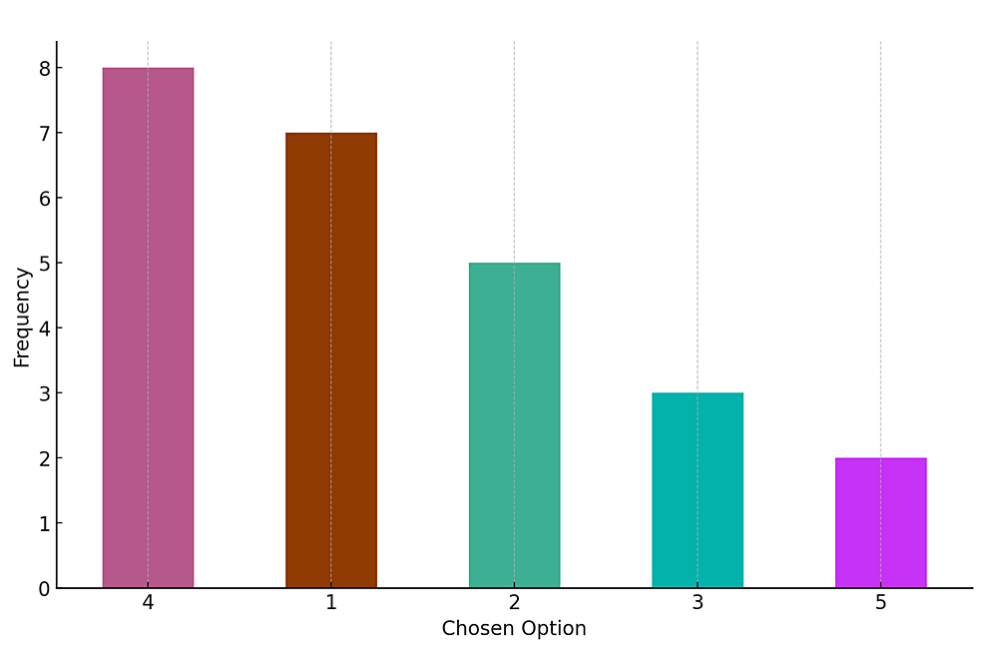
Distribution of responses for Question 1 Correct answer: Option 1 (AI contour) AI: artificial intelligence

The total number of responses was 25. The most chosen option was Option 4, which was selected by eight respondents (32%). The least chosen option was Option 5, with only two responses (8%). Seven (28%) chose the correct option. The graph depicts the distribution of responses (Figure 2).

**Figure 3:**
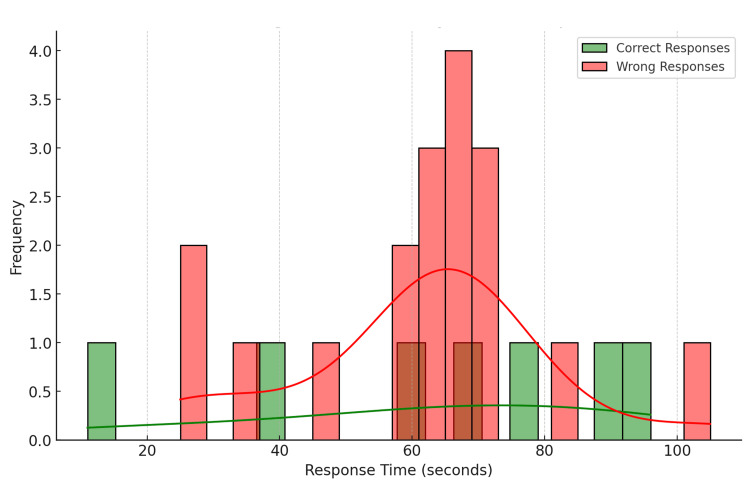
Combined histogram and density plot of response times Question 1 Green: correct responses; Red: wrong responses

Distribution of response times for Question 1 (Figure 3)

The average response time for the survey was approximately 61.48 seconds. Respondents who chose the correct answer (Option 1) had an average response time of 62.14 seconds. Those who chose incorrectly had an average response time of 61.22 seconds. The fastest response time was 11 seconds, while the slowest was 105 seconds. The overlap in response times between correct and wrong answers indicates that the time taken to respond was not necessarily an indicator of a correct or incorrect answer.

Distribution of responses for Question 2: AI contour was Option 2 (correct answer)

**Figure 4:**
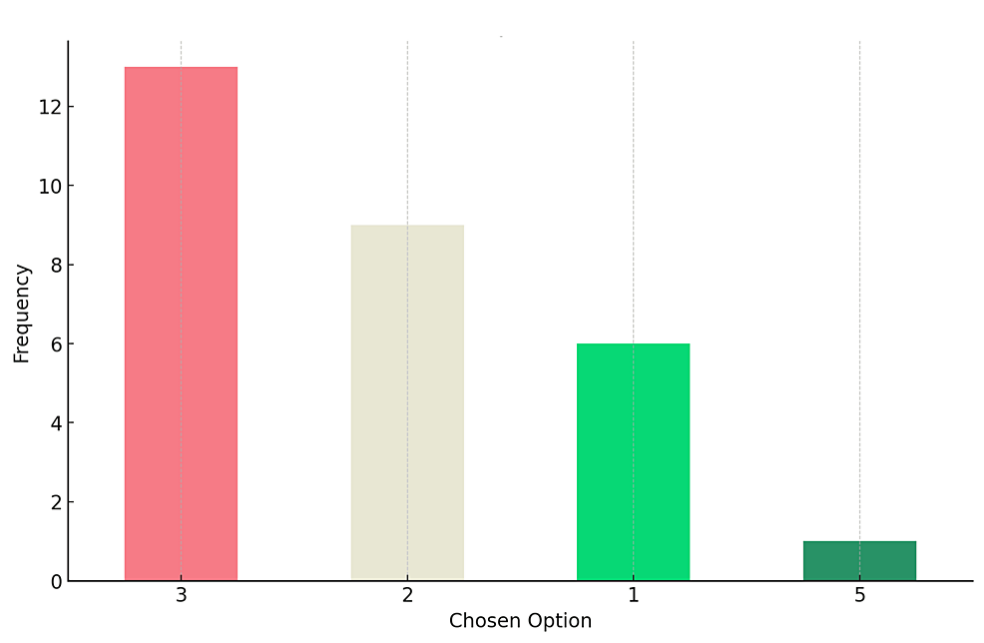
Distribution of responses for Question 2 Correct answer: Option 2 (AI contour) AI: artificial intelligence

There were a total of 29 responses. The most chosen option was Option 3, which was selected by 13 respondents (44.82%). The least chosen option was Option 5, with only one response (3.44%). The correct option was chosen by nine participants (31.03%), and none chose option 4. The graphs depict the distribution of responses (Figure 4).

**Figure 5:**
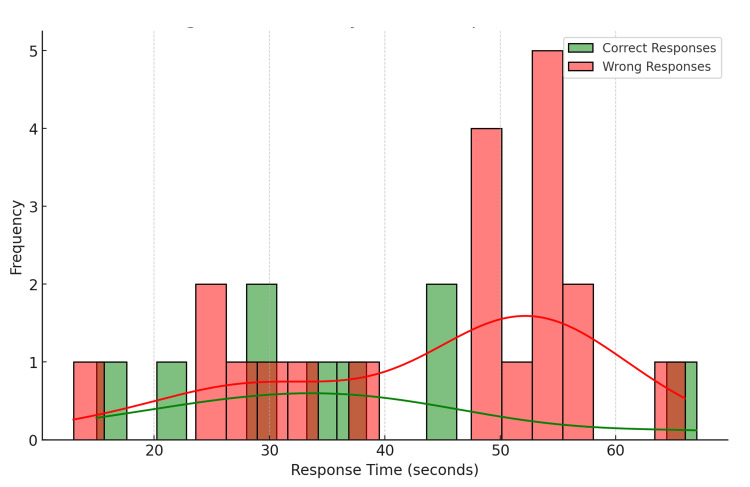
Combined histogram and density plot of response times for Question 2 Green: correct responses; Red: wrong responses

**Figure 6:**
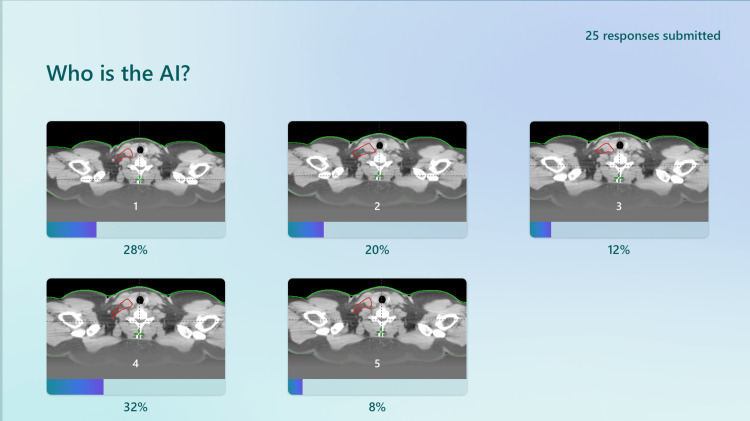
Question 1 The figure is question 1, which has four options: Option 1 is the contour delineated by the artificial intelligence software. Image Credits: Theresa Binz & Nandan Shanbhag (Authors)

**Figure 7:**
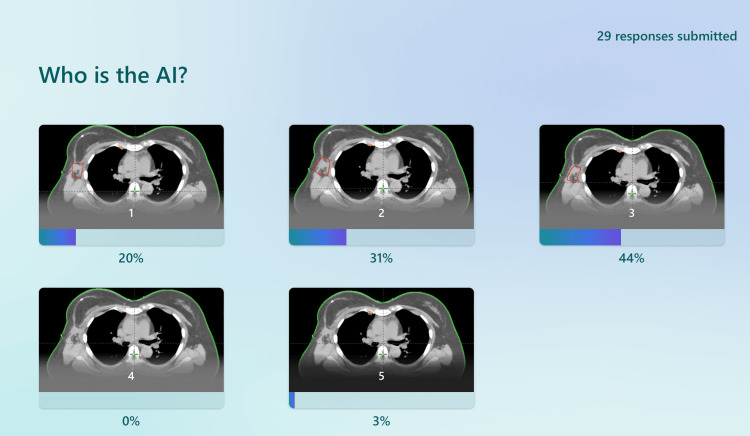
Question 2 The figure is question 2, which has four options: Option 2 is the contour delineated by the artificial intelligence software. Image Credits: Theresa Binz & Nandan Shanbhag (Authors)

Distribution of response times for Question 1 (Figure 5)

The average response time for the survey was approximately 41.69 seconds. Respondents who chose the correct answer (Option 2) had an average response time of 35.89 seconds. Those who chose incorrectly had an average response time of 44.30 seconds. The fastest response time recorded was 13 seconds, while the slowest was 67 seconds.

Upon statistical comparison of correct responses for Questions 1 and 2 analyzed with a chi-square test, a chi-square statistic (Yates correction) of 0.0031 with a p-value of 0.955867 emerged, suggesting no statistically significant difference (higher than threshold p<0.05) in the proportion of correct responses between the two questions. This implies that the participant's ability to identify the AI-generated contours correctly did not significantly change between the first and the second question.

## Discussion

This study presents significant findings regarding the application of AI in radiation oncology, particularly in contouring processes. Notably, AI systems such as Limbus® AI demonstrate an accelerated completion of contouring tasks, substantially exceeding the performance of human oncologists. The duration for AI to complete these tasks is approximately 60 seconds, in contrast to 570 seconds for the fastest human oncologist. This efficiency presents a potential for a substantial reduction in clinical workflow times.

The study further reveals human oncologists' challenges in distinguishing between AI-generated and human-delineated contours. Additional research supports this observation, indicating that AI algorithms enhance contouring quality and efficiency by integrating data with multiple imaging modalities while maintaining accuracy [9].

Variability among ROs in contouring times is attributed to differences in individual approaches and experience levels. This variability is more pronounced in complex anatomical regions, indicating the diverse methodologies in professional practice [10]. There is a noted high consensus among oncologists for simpler structures; however, complex or high-risk areas exhibit considerable variability [11]. This variation is also influenced by the choice of imaging modality, impacting the level of detail available for contouring [12].

Survey outcomes from the study indicate a nuanced understanding of identifying AI-generated contours. A substantial proportion of respondents accurately identified AI-generated contours, yet the choice distribution and response times suggest uncertainty. This is consistent with research showing that AI-generated contours match the accuracy of those produced by expert oncologists in certain organs [13]. AI has also been observed to enhance contouring accuracy, reduce observer variation, and decrease contouring time [14]. Additionally, AI's role in detecting errors in human-generated contours underscores its comprehensive utility in radiation oncology [15].

The importance of ongoing quality control and validation in ensuring AI-generated contours' reliability and clinical utility is highlighted. Integrating data-driven algorithms with deep neural networks is proposed to improve the precision of AI-generated contours [16]. This approach and AI's capability in error detection suggest a synergistic relationship between AI and human expertise in radiation oncology contouring.

Lastly, the perception and acceptance of AI-assisted technologies in radiation oncology are predominantly favorable. Studies indicate a high acceptance rate of AI technology for radiation contouring among oncologists, with minimal resistance to its adoption [17]. This favorable perception is crucial for effectively integrating AI technologies in clinical settings.

Limitations of the study

The study involved a relatively small number of human experts (four ROs), which may not represent the broader population of oncologists with varying levels of experience and expertise. Individual differences in contouring techniques among human oncologists could influence the results. These differences might not necessarily reflect the overall proficiency or accuracy but rather personal or institutional preferences. A larger and more diverse sample of oncologists might provide a more comprehensive comparison. The study focused solely on Limbus® AI. Different AI software might yield different results, limiting the generalizability of the findings to other AI systems. The participants' ability to identify AI-generated contours could be influenced by their preconceived notions about AI capabilities. This might have affected their objectivity in distinguishing between AI and human-generated contours. In the blind comparative analysis, the potential for a learning effect over multiple rounds of evaluation was not thoroughly addressed. Participants might become better at identifying AI-generated contours with more exposure. Only two rounds of questioning were applied.

The study used de-identified post-lumpectomy breast cancer patient CT scans. The complexity of cases can vary, and the results might differ in more complex or different types of cases. The performance of the AI software could be influenced by the technical specifications of the system it was operated on, which might not reflect its capability in different or more advanced setups. The study was a one-time evaluation and did not assess how the performance of both humans and AI might evolve over time with advancements in technology and experience.

## Conclusions

This study demonstrates the integration of AI in radiation oncology, particularly in the contouring process. AI’s ability to expedite the contouring process, often surpassing human oncologists in speed and efficiency, is not just a reflection of rapid technological progress but a transformative development in clinical practice. Its precision and reliability are evident in generating accurate contours, especially in complex anatomical areas and when compared to human-generated contours.

However, the integration of AI into clinical practice requires careful consideration of several factors. The variability in contouring approaches among human oncologists, shaped by individual experience and methodology, highlights the need for a synergistic relationship between AI and human expertise. AI should be seen as a complementary tool, augmenting oncologists’ abilities where its precision and efficiency are most advantageous. Additionally, the positive perception and acceptance of AI-assisted technologies by ROs are vital for their successful implementation, facilitating a smoother transition to an AI-integrated future in radiation therapy. Continuous research, quality control, and validation are crucial to ensure that AI technologies evolve in line with clinical needs and uphold the highest standards of patient care in radiation oncology.
